# The London Classification: Improving Characterization and Classification of Anorectal Function with Anorectal Manometry

**DOI:** 10.1007/s11894-020-00793-z

**Published:** 2020-09-15

**Authors:** S. Mark Scott, Emma V. Carrington

**Affiliations:** 1 Neurogastroenterology Group and GI Physiology Unit, Centre for Neuroscience, Surgery & Trauma, Blizard Institute, Queen Mary University London, London, UK; 2 Surgical Professorial Unit, St Vincent’s University Hospital, Dublin, Ireland

**Keywords:** Anorectal manometry, London Classification, IAPWG protocol, Constipation, Evacuation disorder, Faecal incontinence

## Abstract

**Purpose of Review:**

Objective measurement of anorectal sensorimotor function is a requisite component in the clinical evaluation of patients with intractable symptoms of anorectal dysfunction. Regrettably, the utility of the most established and widely employed investigations for such measurement (anorectal manometry (ARM), rectal sensory testing and the balloon expulsion test) has been limited by wide variations in clinical practice.

**Recent Findings:**

This article summarizes the recently published International Anorectal Physiology Working Group (IAPWG) consensus and London Classification of anorectal disorders, together with relevant allied literature, to provide guidance on the indications for, equipment, protocol, measurement definitions and results interpretation for ARM, rectal sensory testing and the balloon expulsion test.

**Summary:**

The London Classification is a standardized method and nomenclature for description of alterations in anorectal motor and sensory function using office-based investigations, adoption of which should bring much needed harmonization of practice.

## Introduction

In patients with refractory symptoms of faecal incontinence (FI) or constipation/evacuation disorder (ED), who have failed to respond to standard conservative or medical therapies, a number of complementary diagnostic investigations exist for the assessment of anorectal structure and of motor and sensory function [1, 2]. For those in whom advanced management strategies are being considered, such diagnostic testing should be considered a compulsory component of clinical evaluation, as it augments understanding of underlying pathoaetiology, which is often multifactorial, and establishes a physiological diagnosis to which treatments can be more optimally directed. Symptoms alone are poor predictors of response to treatment. Anorectal manometry (ARM), which provides a dynamic measure of intraluminal pressure, is the best established and most widely available investigative tool in the diagnostic armamentarium and enables an objective evaluation of parameters of both anal and rectal function, such as tone, contractility and relaxation, as well as rectoanal coordination and reflex activity and also rectal sensation [1••].

Anorectal manometric techniques have been available for over half a century, and until recently, evaluation of pressure change was achieved through ‘conventional’ ARM, utilizing a limited number of recording sensors (generally 1–6), with data displayed as pressure line traces interpreted separately [3, 4]. The last consensus guidelines for test performance and interpretation of ARM were published almost 2 decades ago, in 2002 [3, 5], and a recent survey of international manometric practice showed absolute failure of consistency between institutions, with no two centres from 107 surveyed in 30 countries describing identical protocols for patient preparation, study setup, protocol and results reporting. Furthermore, no single centre fully adhered to the published guidelines [6•]. This variability has understandably compromised test credibility, clinical interpretation, transfer of data between institutions and research collaboration.

Although conventional ARM remains in use in some centres [6•], manometric technology has greatly advanced over the past decade, with so-called high-resolution (HR-ARM) or three-dimensional high-definition anorectal manometry (3D HD-ARM) now able record and display detailed information simultaneously from the whole anal canal and distal rectum [6, 7]. Improved spatial resolution of data acquisition has been achieved through the use of catheters containing a greater number of closely-spaced recording sensors (typically ≥ 8), together with developments in signal processing. Interpolation between adjacent recording sensors now enables intraluminal pressure to become a spatial continuum; recorded data are hence displayed as colour-contoured pressure topography plots, rather than overlapping line traces, with pressure magnitude indicated by changes on the colour spectrum. Such technology has already been shown to improve diagnostic accuracy in testing of the upper GI tract [8] and has led to a validated diagnostic classification system (The Chicago Classification, now in its third iteration) to aid in the management of oesophageal motility disorders [9]. Development of such a classification system has contributed to better acceptance and standardization of the technique.

Compared with that of the upper gut, uptake of high-resolution techniques for the assessment of anorectal function has been very slow; only 100 original articles were available in the scientific literature at the time of writing (March 2020). Nevertheless, by 2017, the international survey described above confirmed that greater than 50% of institutions had already introduced HR-ARM/3D HD-ARM into their clinical practice [6•]. In a bid to establish consensus and set new minimum standards for HR-ARM (within a broader remit of standardizing the clinical measurement of anorectal function), an international anorectal physiology working group (IAPWG) was established, comprising 29 experts in the field. This group has very recently published a new consensus guideline document [10••] which provides a standardized protocol for the performance of anorectal manometry testing (and is the first to incorporate high-resolution technology), applicable to devices produced by any manufacturer and also introduces the first classification system for disorders of anorectal function based on objective physiological measurements (the London Classification). This manuscript will focus on how the protocol and classification system can be used to improve diagnosis in anorectal disorders.

## Clinical Indications

Manometric assessment of anorectal function is indicated in patients in whom organic pathology has been appropriately excluded and in whom the cause of their intractable symptoms remains elusive [2, 11]. Manometry should not be performed in isolation and should, as a minimum, be accompanied by the assessment of rectal sensation and a direct test of evacuation (e.g. defaecography or the balloon expulsion test). Full evaluation may involve other complementary investigations (e.g. endo-anal ultrasound, gut transit studies, etc.).

ARM provides the opportunity to evaluate several functions of the anorectum, namely, (1) recto-anal reflex activity, (2) anal sphincter function, (3) recto-anal coordination during simulated defecation and (4) rectal sensory function [1, 10]. According to the standardized investigation protocol (10), this is achieved through the sequential performance of a number of pre-defined manoeuvres:

- A period of rest, to evaluate basal anal resting tone
- Voluntary anal squeeze manoeuvres, to evaluate anal contractility
- A cough manoeuvre, to evaluate the anorectal reflex response (as well as sphincteric ‘reserve’ function)
- A simulated defecation, or ‘push’ manoeuvre, to assess variables (rectal propulsive pressure and anal response) deemed to be relevant to the process of defecation
- Bolus (rapid) rectal distension, to evaluate the recto-anal inhibitory reflex
- Progressive rectal distension, to evaluate rectal sensation
- A test of evacuation, to complement the simulated defecation manoeuvre above

The principal indications for ARM are [10, 12]:

1. Assessment of symptoms of FI: primarily for identification/quantification of impaired anal sphincter function (hypotension and/or hypocontractility) and abnormal rectal sensitivity (both heightened [hyper-] and diminished [hyposensitivity])
2. Assessment of symptoms of constipation/ED: primarily for identification / quantification of abnormalities of recto-anal coordination (during ‘push’ [simulated defecation]) and rectal sensitivity (particularly diminished rectal sensation [hyposensitivity])
3. Assessment of symptoms of functional anorectal pain: primarily for identification/quantification of elevated anal sphincter tone (hypertension) and abnormalities of recto-anal coordination (during a simulated defecation manoeuvre])
4. Pre-operative assessment of anorectal function: primarily for description of anal sphincter function and recto-anal coordination (during simulated defecation), particularly if intervention is associated with risks to continence (e.g. fistulotomy, lateral sphincterotomy) or ability to evacuate (e.g. rectopexy)
5. Assessment of anorectal function in patients after obstetric injury/traumatic birth: primarily if the clinician and patient wish to quantify anal sphincter function prior to planning of future deliveries
6. To facilitate biofeedback training: primarily to identify/quantify changes in anal function, recto-anal coordination (during simulated defecation) or rectal sensitivity in response to the intervention
7. To quantify the effects of other therapeutic interventions.

## Equipment

Currently, 3 principal HR-ARM systems are available: (1) the ManoScan™ AR manometry system (Medtronic; Minneapolis, Minnesota, USA); (2) the Solar GI manometry system (Laborie, Mississauga, Ontario, Canada); and (3) the InSIGHT manometry system, with Bioview analysis software (Diversatek™, Milwaukee, Wisconsin, USA). Pressure transduction can be achieved by coupling these systems to thin (<5 mm), flexible high-resolution solid-state or water-perfused catheters, containing 8–12 sensors or recording ports, spaced 6–10 mm apart. Conversely, 3D high-definition recordings are achieved using a rigid probe (ManoScan™ AR 3D catheters: Medtronic: 100 mm length × 10.75 mm diameter) housing 256 individual pressures sensors arranged in a 16 × 16 grid (i.e. 16 rows spaced 4 mm apart, each containing 16 circumferentially oriented sensors 2.1 mm apart).

More recently, air-charged catheters have become available (e.g. T-DOC®; Laborie: Mississauga, Ontario, Canada); however, these are currently limited to 4–6 sensors, which would be considered conventional ARM. Further technical specification of commercially available catheters and manometry systems can be found elsewhere [7, 13]. For all catheter types, a non-latex balloon (≥ 3.3 cm long, with minimum capacity of 400 mls) should be secured to the proximal tip for sensory testing [7.13].

A further recent innovation is the availability of ‘bedside’ manometry through portable equipment (e.g. mcompass: Medspira; Minnesota, USA, to which an air-charged catheter is attached [14]; Anopress: THD Worldwide, to which a single-channel sleeve catheter is linked [15]; or a wireless catheter (Ningbo Maida Medical Device Inc.: Ningbo, China [16]). At present, ‘bedside’ HR-ARM is only feasible using the Ningbo Maida Medical system.

## The International Anorectal Physiology Working Group (IAPWG) Protocol

**Fig. 1:**
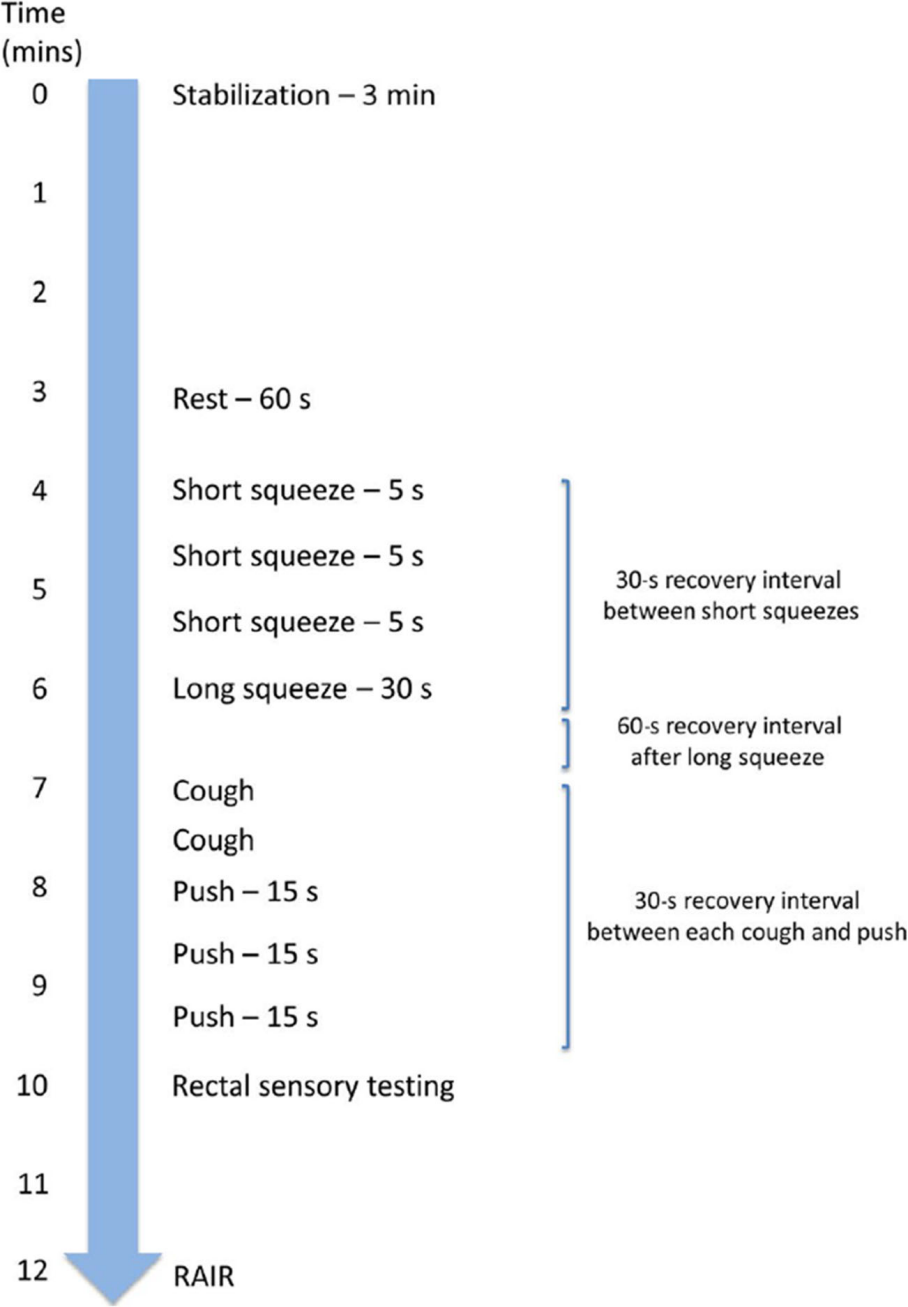
**Schematic of the standardized IAPWG manometry protocol**

A protocol for ARM (Fig. 1a) (with recommended measurements for results reporting [Table 1]) has been recommended by the IAPWG [10••] and consists of the following standardized sequential elements:

1. **Stabilization period**: following catheter insertion and prior to test manoeuvres, a 3-min period of stabilization should be observed to allow anal tone to return to baseline after intubation.
  *No measurements are reported for this manoeuvre*.
2. **Rest**: this is the manoeuvre that measures basal anal tone at rest and is recorded over 60 s.
  *Quantitative measurement of anal resting pressure is reported, as well as a qualitative description of ultra-slow waves, if present* [17].
3. **(Short) squeeze**: this is the manoeuvre that records anal pressure during voluntary effort to contract the anus/pelvic floor. Three squeezes are performed, each of 5 s duration, separated by 30-s between-manoeuvre recovery intervals. The best (defined as the most qualitatively normal) attempt should be used for analysis.
  *Quantitative measurement of anal squeeze pressure is reported (maximum incremental pressure)*.
4. **Long (endurance) squeeze**: this is the manoeuvre that records anal pressure during sustained voluntary effort over 30 s. The principal aim is to describe fatigue over time rather than purely contractile ability, as measured during ‘(short) squeeze’ (above). A single endurance squeeze is performed followed by a 60-s between-manoeuvre recovery interval.
  *Quantitative measurement of endurance squeeze pressure is reported*.
5. **Cough**: this is the manoeuvre that measures recto-anal pressure changes during cough, i.e. assesses the reflex increase in anal sphincter pressure during an abrupt change in intra-rectal (surrogate of intra-abdominal/intra-pelvic) pressure. Two single coughs are performed, separated by a 30-s between-manoeuvre recovery interval. The best attempt (defined as the attempt associated with the greatest increase in rectal pressure) is used for analysis.
  *Quantitative measurement of both rectal pressure during cough and anal pressure during cough is reported (maximum pressure change recorded)*.
6. **Push**: this is the manoeuvre that measures anal and rectal pressure changes during simulated defecation. Three pushes are performed, each of 15 s duration, separated by 30-s between-manoeuvre recovery intervals. The best (defined as the most qualitatively normal) attempt should be used for analysis.
  *Quantitative measurement of the rectal pressure change during push and the anal pressure change during push are reported*. The authors acknowledge that analysis of the push manoeuvre is often subject to confusion. Although the ‘expected’ anal pressure change during push is a relaxation (and hence a negative recorded value, when referenced to the maximum anal pressure recorded during the period of rest immediately preceding the push manoeuvre) [18], this is often not observed [19–21]; indeed, an *increase* in anal pressure (from the anal pressure recorded immediately prior to the push manoeuvre) may be seen and hence a positive value recorded. The distinction between this and a ‘paradoxical contraction’ (abnormal finding) needs to be appreciated.
7. **Recto-anal inhibitory reflex (RAIR)**: this is the manoeuvre which measures reflex anal response to rapid rectal distension. A single RAIR manoeuvre is initially performed with a starting volume of at least 30 mls. However, it should be noted that failure to elicit the RAIR may be seen with low distending volumes in a large capacity rectum. If megarectum is suspected, the test should be repeated with progressively larger volumes of air (e.g. incrementally in 50 ml aliquots). The RAIR is followed by a 30-s recovery interval.
  *Qualitative measurement is reported as the recto-anal inhibitory reflex (with a normal response characterized by an anal pressure decrease during rectal balloon distension)*.
8. **Rectal sensory test**: this is the procedure that assesses rectal sensitivity to distension utilizing the balloon attached to the catheter tip. The IAPWG protocol does not mandate adherence to either ramp (continuous) or (intermittent) phasic distension techniques; therefore either can be used. For ramp distension, a rate of 1–5 mL/s should be employed, and for phasic distension, the inflation rate should be set at 10 mL/s.
  *Quantitative measurement of balloon volume is recorded for each of the three patient-reported sensory thresholds: first constant sensation volume (FCSV), desire to defecate volume (DDV) and maximum tolerated volume (MTV)*. *A fourth sensory threshold (sustained urgency volume) is optional.*
9. **Balloon expulsion test or defecography**: the utility of ARM alone, for diagnosing disorders of rectoanal coordination, is uncertain [21••]; accordingly, the London Classification requires the results of an ARM study to be considered *in conjunction* with those of a direct test of evacuation (i.e. the balloon expulsion test or defaecography) [22•]. The balloon expulsion test is the procedure that assesses the individual’s ability to expel a rectal balloon filled with 50 ml of water whilst in the sitting position. Alternatively, defecography assesses the individual’s ability to expel neostool (contrast medium) whilst upright on a commode (barium defecography) or supine (magnetic resonance defecography).
  *Quantitative measurement is reported as the balloon expulsion time or quantity/time taken for neostool expulsion, respectively*.

**Table 1:** Recommended measurements for anorectal manometry, the balloon expulsion test and the rectal sensory test

Test	Manoeuvre	Measurements	Definition	Included in London Classification	Measurement type		Units
					Quantitative	Qualitative	
ARM	Stabilization	N/A	Period of 3 min to allow stabilization of anal resting tone	NO	N/A	N/A	N/A
	Rest	Anal resting pressure	Mean maximum pressure measured from the whole anal canal over a 60 s recording period	YES	X		mmHg
		Ultra-slow waves	The presence of repeated pressure oscillations within the anal canal, occuring at 0.5–2 min^−1^	NO		X	present / absent
	Squeeze	Anal squeeze pressure	Maximum incremental pressure observed during the 5 s short squeeze		X		mmHg
	Long squeeze	Endurance squeeze pressure	The duration of time the subject under study can voluntarily sustain an increase in anal pressure > 50% of maximum incremental squeeze pressure during the 30 s long squeeze	NO	X		secs
	Push	Rectal pressure change during push	Maximum pressure change recorded within the rectum during the push manoeuver	YES	X		mmHg
		Anal pressure change during push	Maximum pressure change recorded within the anal canal during the push manoeuver	YES	X		mmHg
	Cough	Rectal pressure during cough	Maximum pressure change recorded within the rectum during cough manoeuver	NO			mmHg
		Anal pressure during cough	Maximum pressure change recorded within the anal canal during the push manoeuver	NO	X		mmHg
	RAIR	Rectoanal inhibitory reflex	Reflex reduction in maximum anal pressure in response to rapid distension of the rectum	YES		X	present / absent^a^
BET^b^	Expulsion	Balloon expulsion time	Time taken in seconds to expel a rectal balloon	YES	X		secs^c^
RST	Rectal sensory thresholds^d^	First sensation volume	The minimum balloon insuflation volume required to elecit a sensory	YES	X		mls
		Desire to defaecate volume	The balloon insufflation volume required to elicit a sustained desire to defaecate	YES	X		mls
		Maximum tolerated volume	The balloon insufflation volume that causes an intolerable desire to defaecate	YES	X		mls

*ARM*  anorectal manometry, *BET* balloon expulsion test, *RST*  rectal sensory test

N/A = not applicable.

^a^The volume required to elicit the RAIR should also be documented

^b^Alternate test is defecography

^c^The presence or absence of the desire to defaecate during the procedure should also be documented

^d^Sustained urgency volume threshold is optional and defined as the balloon insufflation volume required to elicit a sense of faecal urgency

For quantitative assessment of an ARM study, automated calculation of a number of user-defined measures is achieved through the use of proprietary software. Recorded values can then be referenced to normative datasets appropriate to the equipment used (as normal values are not currently interchangeable between technologies [23•]). Several large datasets (> 100 healthy subjects) now exist for HR-ARM [24–26] and 3D-HDAM [27, 28], as well as conventional ARM [29, 30], which can be employed in clinical practice to define abnormalities of individual test manoeuvre results and hence to aid provision of a manometric diagnosis (through population of the London Classification, see below). It must be noted, however, that to date, no normative datasets specifically utilizing the full IAPWG protocol exist in the literature.

## The London Classification for Disorders of Anorectal Function

**Fig. 2:**
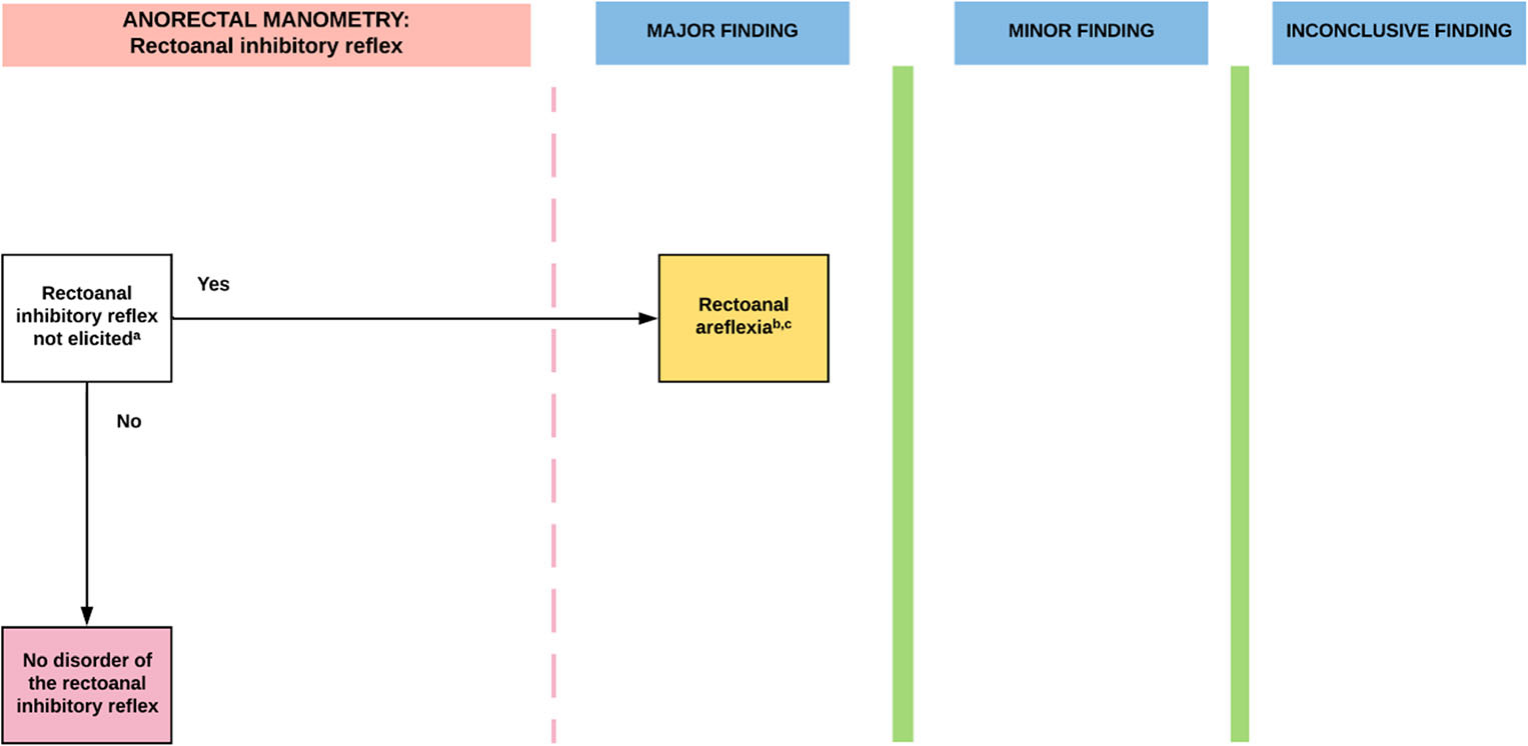
**IAPWG classification Part 1**: *Disorder of the rectoanal inhibitory reflex*. For this and subsequent figures, the diagrams are colour-coded for clarity: (i) white boxes represent manometric findings or decision points; (ii) yellow boxes represent the resultant diagnosis; and (iii) pink boxes represent a ‘negative/normal’ study. ^a^Minimum volume required to elicit reflex not established in the literature: failure to elicit a RAIR may be seen with low distending volumes in a large capacity rectum. ^b^RAIR not elicited is a pattern not seen in health but may be found in asymptomatic patients following rectal resection/ileal pouch anal anastamosis, anal hypotonia, faecal loading or megarectum. ^c^May indicate the need for further investigation to exclude aganglionosis especially in paediatric populations and adult patients with co-existent megarectum/megacolon. All results to be interpreted in the context of adjunctive testing

**Fig. 3:**
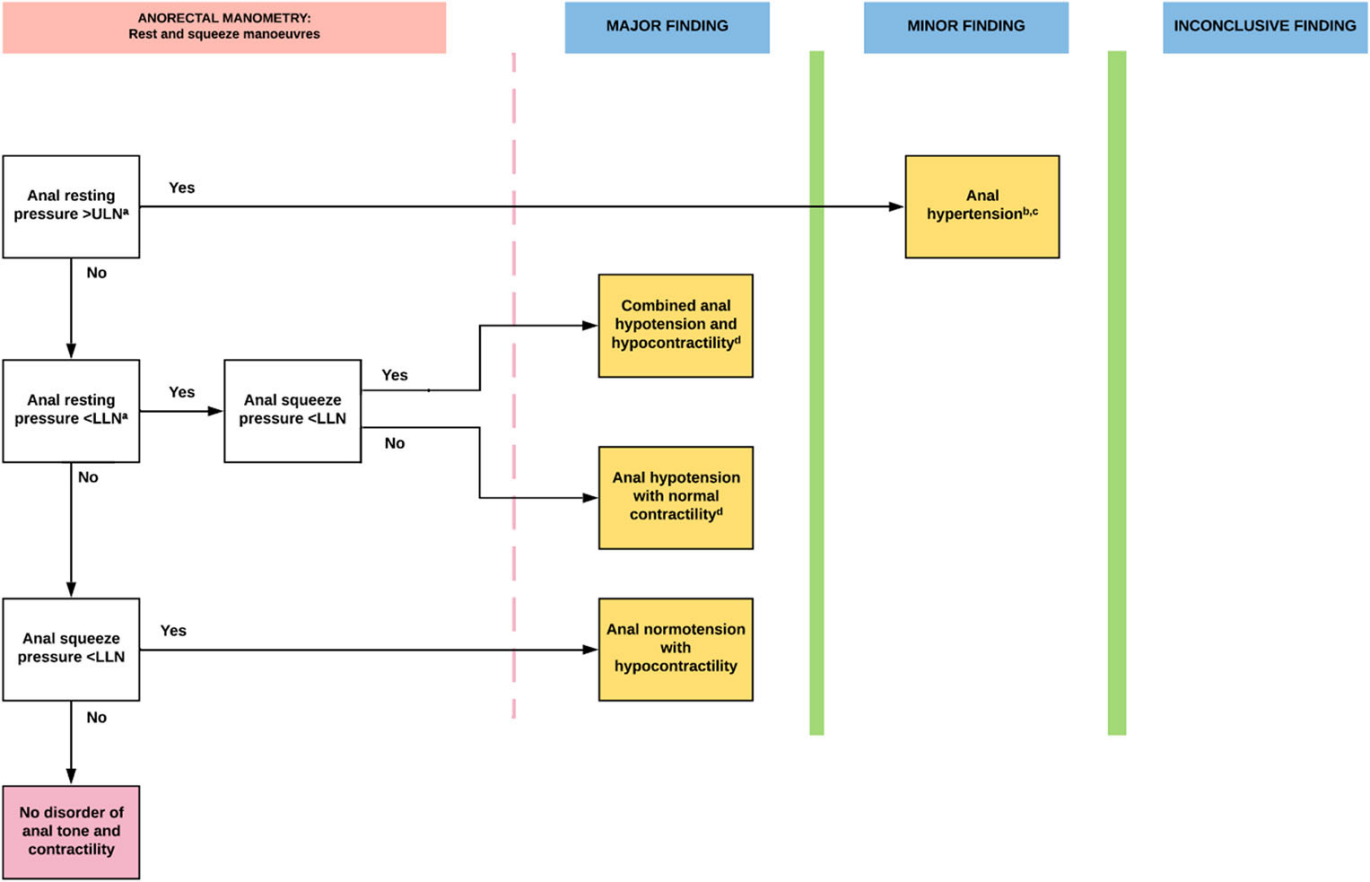
**IAPWG classification part 2**: *Disorders of anal tone and contractility*. ^a^The functional anal canal length may be measured, as a short anal canal can be associated with anal hypotonia, but its use as a diagnostic criterion in isolation is unproven. ^b^It may be associated with slow and/or ultraslow waves; however the clinical significance of these has not been established. ^c^This finding may have greater clinical significance in certain patient groups (e.g. chronic anal fissure, levator ani syndrome or proctalgia fugax). ^d^Addition of an abnormal cough response may indicate a more severe phenotype (whereas preservation may suggest a target for biofeedback), but its use as a diagnostic criterion is unproven. All results to be interpreted in context of adjunctive testing. *LLN* Lower limit of normal ULN

**Fig. 4:**
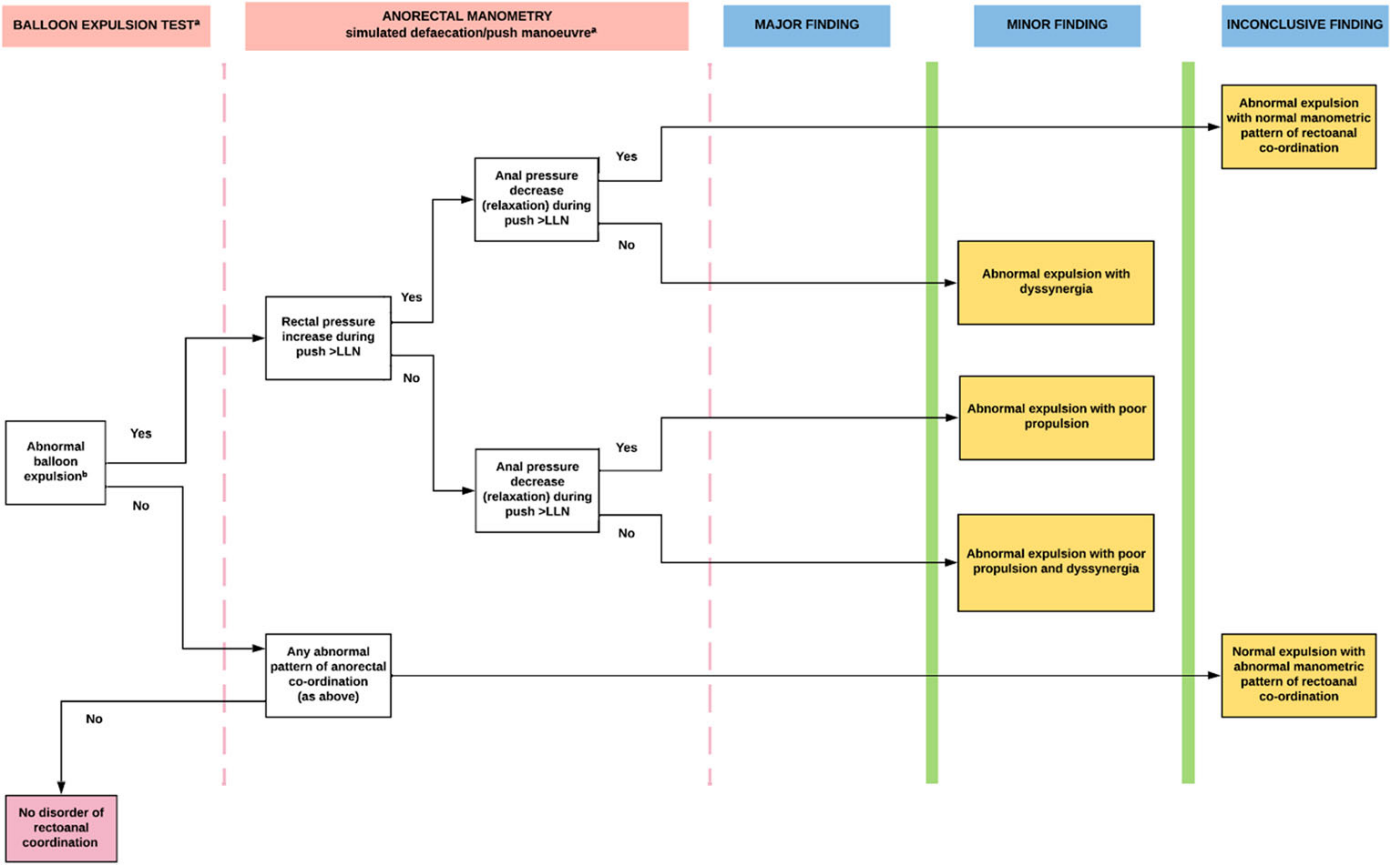
**IAPWG classification part 3**: *Disorders of rectoanal coordination*. ^a^It requires the use of both balloon expulsion test and anorectal manometry ^b^or impaired evacuation of contrast medium (prolonged evacuation end time and/or reduced percentage of contrast emptied) on alternative testing, e.g. barium or MR defecography. All results to be interpreted in context of adjunctive testing. * akin to ‘type I’ dyssynergia. ** akin to ‘type IV’ dyssynergia. *** akin to ‘type II’ dyssynergia. *LLN* Lower limit of normal ULN

**Fig. 5:**
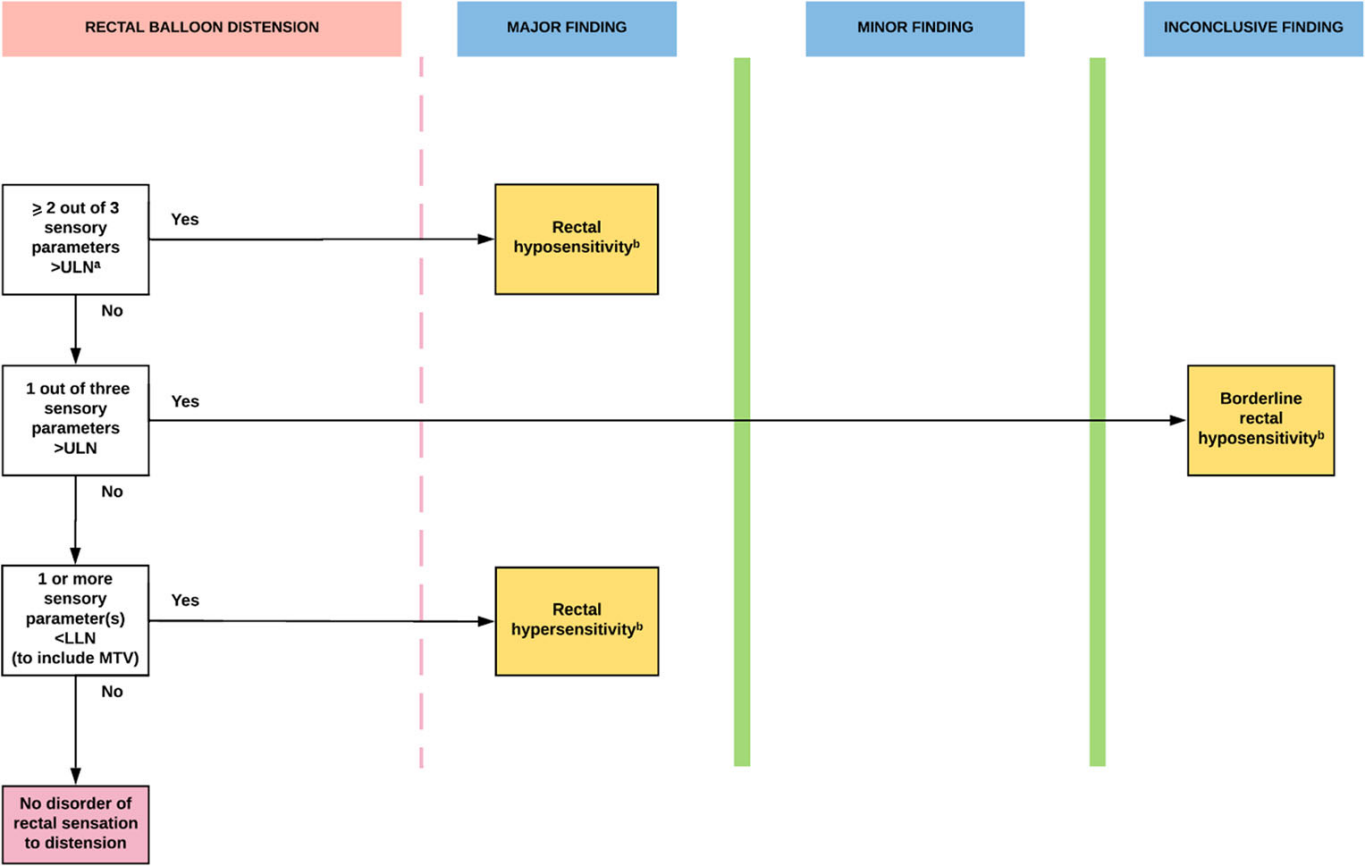
**IAPWG classification part 4**: *Disorders of rectal sensation*. ^a^Sensory parameters are first constant sensation volume (FCSV), desire to defecate volume (DDV) and maximum tolerated volume (MTV). ^b^Abnormal results may be further described using additional methods (e.g. barostat to assess compliance). All results to be interpreted in context of adjunctive testing. *LLN* Lower limit of normal ULN

This is the first classification system for the diagnosis of disorders of anorectal function, based on objective measurements from a manometry study. Utilizing the IAPWG protocol, the measurements described above are required to populate each of the 4 parts of the London Classification (Figs. 2, 3, 4, 5) with each of the 4 parts being reported for a single study [10••].

**Fig. 6:**
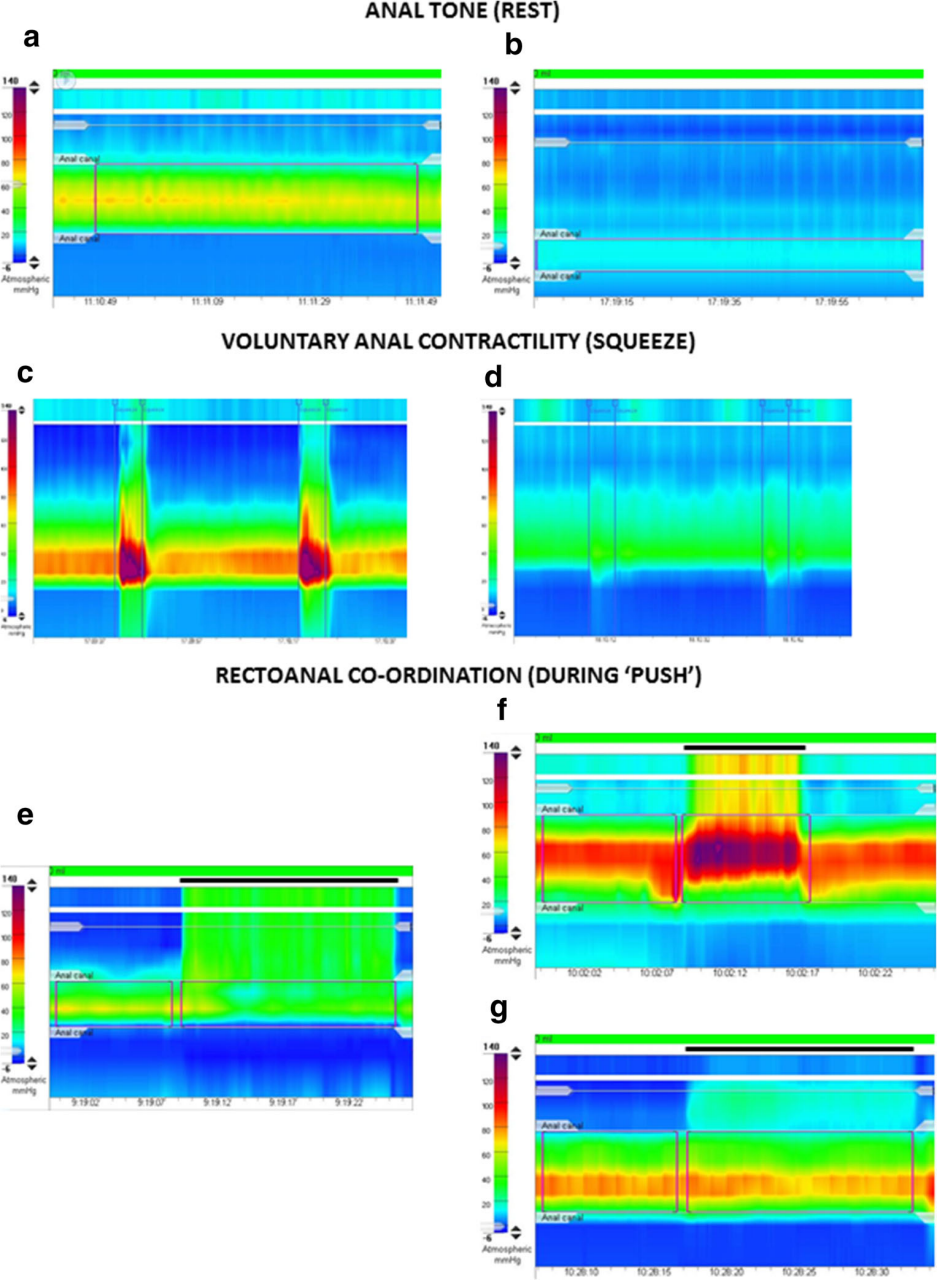
**Anorectal manometric abnormalities**. In this figure, examples of high-resolution manometry colour-contour plots are shown of the individual disorders as classified in the London Classification. *Anal tone (rest)—*1 min period: (**a**) normotonia (mean 65 mmHg) and (**b**) anal hypotonia (mean 17 mmHg). *Voluntary anal contractility (squeeze)*—2 short (5 s) squeezes shown: (**c**) normal anal contractility and (**d**) anal hypocontractility. *Rectoanal coordination (during ‘push’)*—manoeuvre period marked by thick black line: (**e**) normal rectoanal coordination, good rectal propulsion effecting a positive recto-anal pressure gradient (rectal pressure always exceeding anal pressure during the manoeuvre); (**f**) anal dyssynergia, marked increase in anal pressure, so that anal pressure is higher than rectal pressure at all time-points during the manoeuvre (i.e. the recto-anal pressure gradient is negative); and (**g**) poor rectal propulsion, the recto-anal pressure gradient is again negative

Resultant diagnoses for each part of the classification are as follows:

1. **Disorder of the rectoanal inhibitory reflex** (Fig. 2)*. Manometric diagnosis*: ***the*** absence of a RAIR is termed **rectoanal areflexia**.
2. **Disorders of anal tone and contractility** (Fig. 3). *Manometric diagnoses:* **anal hypotension** (Fig. 6a) or **anal hypertension** describes reduced or increased anal resting pressure, respectively. **Anal hypocontractility** (Fig. 6d) describes reduced anal squeeze pressure. **Combined anal hypotension and hypocontractility** describes a co-existent reduction in both anal resting pressure and anal squeeze pressure.
3. **Disorders of rectoanal co-ordination** (Fig. 4). The current London Classification requires the results of *both* the push manoeuvre and either the balloon expulsion test or defecography to be considered in combination. *Manometric diagnoses:* **abnormal expulsion with dyssynergia** (Fig. 6f) describes prolonged expulsion with a positive anal pressure change (anal contraction) greater than that seen in health. **Abnormal expulsion with poor propulsion** (Fig. 6g) describes prolonged expulsion with a reduced rectal pressure change. **Abnormal expulsion with both poor propulsion and dyssynergia** describes prolonged expulsion with both a reduced rectal pressure change and positive anal pressure change (anal contraction) greater than that seen in health. **Normal expulsion with abnormal manometric pattern of rectoanal coordination** describes any of the 3 push findings described above in the presence of normal expulsion. **Abnormal expulsion with normal manometric pattern of rectoanal co-ordination** describes prolonged expulsion in the presence of a normal rectal pressure change and normal anal pressure change.
4. **Disorders of rectal sensation** (Fig. 5). *Manometric diagnoses:* **rectal hyposensitivity** (≥ 2 thresholds above the upper limit of normal) or **borderline rectal hyposensitivity** (1 threshold above the upper limit of normal) describes diminished rectal sensation. **Rectal hypersensitivity** (≥ 1 sensory threshold, including MTV, below the lower limit of normal) describes heightened rectal sensation.

Similar to the Chicago classification of oesophageal motility disorders [9], the clinical relevance of diagnoses is indicated by the hierarchical division of findings into:

- **Major findings**: this is a pattern not seen in healthy control subjects and is likely to represent a physiological alteration associated with symptom generation. Major findings are (1) rectoanal areflexia, (2) anal hypotension with normal contractility, (3) anal normotension with anal hypocontractility, (4) combined anal hypotension and hypocontractility, (5) rectal hyposensitivity and (5) rectal hypersensitivity.
- **Minor findings**: this is a pattern that is seen in patients with anorectal symptoms, and however, may also be seen in healthy control subjects and may represent a physiological alteration associated with symptom generation. Minor findings are (1) anal hypertension, (2) abnormal expulsion with poor propulsion, (3) abnormal expulsion with dyssynergia and (4) abnormal expulsion with poor propulsion and dyssynergia.
- **Inconclusive findings**: this is a pattern that is seen in patients with anorectal symptoms, but also seen in control subjects. Such findings may be associated with symptom generation, though the relevance is yet to be fully determined. Inconclusive findings according to the London Classification are: (1) abnormal expulsion with normal manometric pattern of recto-anal coordination, (2) normal expulsion with abnormal manometric pattern of recto-anal coordination and (3) borderline rectal hyposensitivity.

Overall, this new framework provides a common language with which to describe manometric results and should be viewed as complimentary to existing disease classifications such as the Rome classification [31]. Although the anorectal disorders section of Rome defines disease entities based on a combination of symptoms and limited physiological findings, it provides little practical advice on how to interpret and report abnormal results.

One notable change in nomenclature is the simplification of terms used for reporting of disorders of rectoanal coordination. Instead of numerical subtypes to describe manometric patterns during the ‘push’ manoeuvre (types I–IV dyssynergia), a combination of two descriptive terms is used: *propulsion* to describe the adequacy of increase in rectal pressure during ‘push’ and *dyssynergia* to describe the finding of (abnormal) anal contraction. Furthermore, any abnormal manometric finding needs to be in conjunction with *impaired evacuation* during balloon expulsion/defecography to be deemed of clinical relevance. This was employed, in part, following a milestone study in which manometry traces were subject to blinded multi-observer analysis [21••] which demonstrated that ARM could not discriminate between healthy control subjects and patients with constipation on the basis of traditional dyssynergic subtypes (types I–IV) [18].

It should also be noted that due to lack of scientific evidence, the current iteration of the London Classification does not incorporate measures derived from the long (30 s) squeeze manoeuvre or the cough manoeuvre. Nevertheless, recent 3D HD-ARM studies have shown that sustained voluntary contraction (long squeeze) is the most discriminant parameter to differentiate constipated and incontinent patients [32]. Similarly, other measures, consistently reported using conventional manometry studies, have not been considered essential by the IAPWG [10••], including functional anal canal length [33], anal relaxation during push and sustained urgency volume during the rectal sensory test. Further, the recto-anal pressure gradient (RAPG) during push has not been incorporated. This is subtraction of the minimum anal pressure from the maximum rectal pressure over the course of the push manoeuvre [19]. A positive value (i.e. rectal pressure exceeding anal pressure) is theoretically expected, but with ‘paradoxical’ contraction, a negative value will be recorded (i.e. anal pressure exceeding rectal pressure).

## Limitations of the Current London Classification

The London Classification only considers certain measures derived from a manometry study together with the ‘functional’ results of a direct test of evacuation. For an individual patient, a more complete and definitive diagnosis requires these results to be contextualized alongside those of other investigations of anorectal structure and function. As the factors contributing to the pathophysiology of both FI and constipation/ED are often multiple and inter-related (indeed a common pathophysiology likely explains the frequent co-existence of these conditions) [34, 35], no single test can be expected to fully characterize relevant abnormalities. By way of example, in a patient with passive faecal incontinence, the finding of anal hypotonia on manometry may be complemented by the finding of a major internal anal sphincter defect on manometry. Conversely, another patient presenting with primary symptoms of passive faecal incontinence may have normal anal sphincter function and structure but have rectal hyposensitivity (as per the London Classification) allied to a megarectum found on defecography (where the incontinence is ‘overflow’). Clearly management approach will differ between these patients. As yet, however, no widely accepted consensus exists which uses the findings from combined anorectal investigations to broadly describe clinical phenotypes.

It must be acknowledged that the IAPWG protocol and London Classification are principally based on the coalescence of expert opinion, rather than direct clinical evidence (which is lacking for many of the components); therefore the recommendations should be considered as a proposed approach and not as validated scientific methodology. Nevertheless, improvement in clinical practice can only begin from a common starting point, and the consensus document which presents the standardized protocol and classification system reflects that sentiment [10••]. Future studies using this methodology will be required to validate its feasibility, duration, timing and practicality.

## Future Considerations

It should be appreciated that the test manoeuvres incorporated in the IAPWG protocol/London Classification (rest, squeeze, push, etc.) were derived from those commonly used during conventional manometry and all have been in use for several decades. However, they may not best describe all aspects of anorectal function. For example, the push manoeuvre does not evaluate evacuation per se, only measures / features deemed to be biologically relevant to the act of defecation. Likewise, there is little evidence to support the enduring assumption that individuals voluntarily squeeze their anal canal during normal deferral of defecation nor evidence to support that this behaviour is altered in incontinence. Voluntary anal squeeze is measured over a period of 5–30 s; however, continent individuals are able to overcome the urge to defecate for much longer than this. Refinement of some existing manoeuvres or the development of novel metrics is required to improve diagnostic utility.

With reference to the latter, several new HR-ARM or 3D HD-ARM parameters and analysis methods have recently been introduced. One advantage of 3D HD-ARM over other manometric methods is its ability to define functional anatomy of the anal canal. Recent work from the USA has illustrated a high degree of pressures asymmetry within the anal canal in health [36]. This has led the authors to suggest a predictive model to distinguish patients with FI from control subjects using automated analysis of the results of 3D HD-ARM studies. Using a combination of pressure values, ‘shape characteristics’, high-pressure zone area and reflective symmetry values, they were able to discriminate between 24 patients and 24 volunteers, with an AUC of 1.0 [37•].

Conversely, refining analysis metrics has also been shown to improve diagnostic utility in patients with FI. Data from the United Kingdom has shown that a novel HR-ARM parameter, the ‘contractile integral’ (which integrates the product of mean pressure increase, sphincteric length and voluntary contraction duration) improves sensitivity of detection of anal hypocontractility from 32 to 55% when compared with maximum anal squeeze increment, as measured by conventional ARM [38].

In studies of constipated patients, a Korean group has extrapolated analysis concepts routinely utilized in HR oesophageal manometry recordings to derive a ‘three-dimensional integrated pressurised volume’ (IPV) calculation (akin to the distal contractile integral [DCI]) [39], which describes the coordination of anorectal activity during simulated defecation [40]. IPV pressure ratio between the upper 1 cm and lower 4 cm of the anal canal during push was found to be significantly more effective in predicting the results of the balloon expulsion test in 204 constipated male patients than conventional measures (RAPG) (receiver operator curve area under curve, 0.74, 95% CI: 0.67 to 0.80; vs. 0.60, 95% CI: 0.52–0.67) [41]. However, others from Europe have found no difference in IPV ratio between asymptomatic and constipated subjects [42]. An alternative approach [43••], using a principal components analysis, was shown to distinguish between patients with a normal and abnormal balloon expulsion test with a sensitivity of 75% (when specificity was set at 75%). However, such complex analyses may not be readily transferable to routine clinical practice.

Other pressure morphologies that may be readily observable on HR-ARM/3D HD-ARM recordings include transient anal sphincter relaxations [24] and differential voluntary contraction morphologies (which allow an assessment of the contribution of external anal sphincter and puborectalis contraction) [24, 44], as well as markers of descending perineum syndrome [45] and rectal intussusception or prolapse [46–48].

Though none of these parameters/analysis methods are yet accepted in current clinical practice, they represent relevant measures that may allow for a redefinition of anorectal anatomy and physiology and possible incorporation within future iterations of the IAPWG protocol/London Classification.

## Summary and Conclusions

The IAPWG protocol and the London Classification provide a standardized method and nomenclature for description of alterations in anorectal motor and sensory function using office-based investigations and are the first collaborative guidance applicable to high-resolution anorectal manometry. This represents a landmark step in standardizing diagnosis of patients presenting with symptoms of anorectal dysfunction. Nevertheless, prospective studies to determine uptake and clinical utility are awaited, with the goal of assessing whether the protocol and classification system positively impact patient management.

Further development is ongoing, with the plan to incorporate results from other, complementary standardized investigations (e.g. endo-anal ultrasound, gut transit studies, defecography, etc.) to provide an evidence-based diagnostic classification system of clinical (patho)physiological phenotypes. Serial diagnostic and outcome studies will then be required to assess the clinical utility of the system for the direction of specific behavioural, medical and surgical interventions.
